# Supplementary material for: Glycine acylation and trafficking of a new class of bacterial lipoprotein by a composite secretion system
Source: eLife. 2021 Feb 24;10:e63762. doi: 10.7554/eLife.63762 (PMC7943197; doi:10.7554/eLife.63762)
Supplement: Figure 2—source data 1. [file elife-63762-fig2-data1.pptx]

## Slide 1
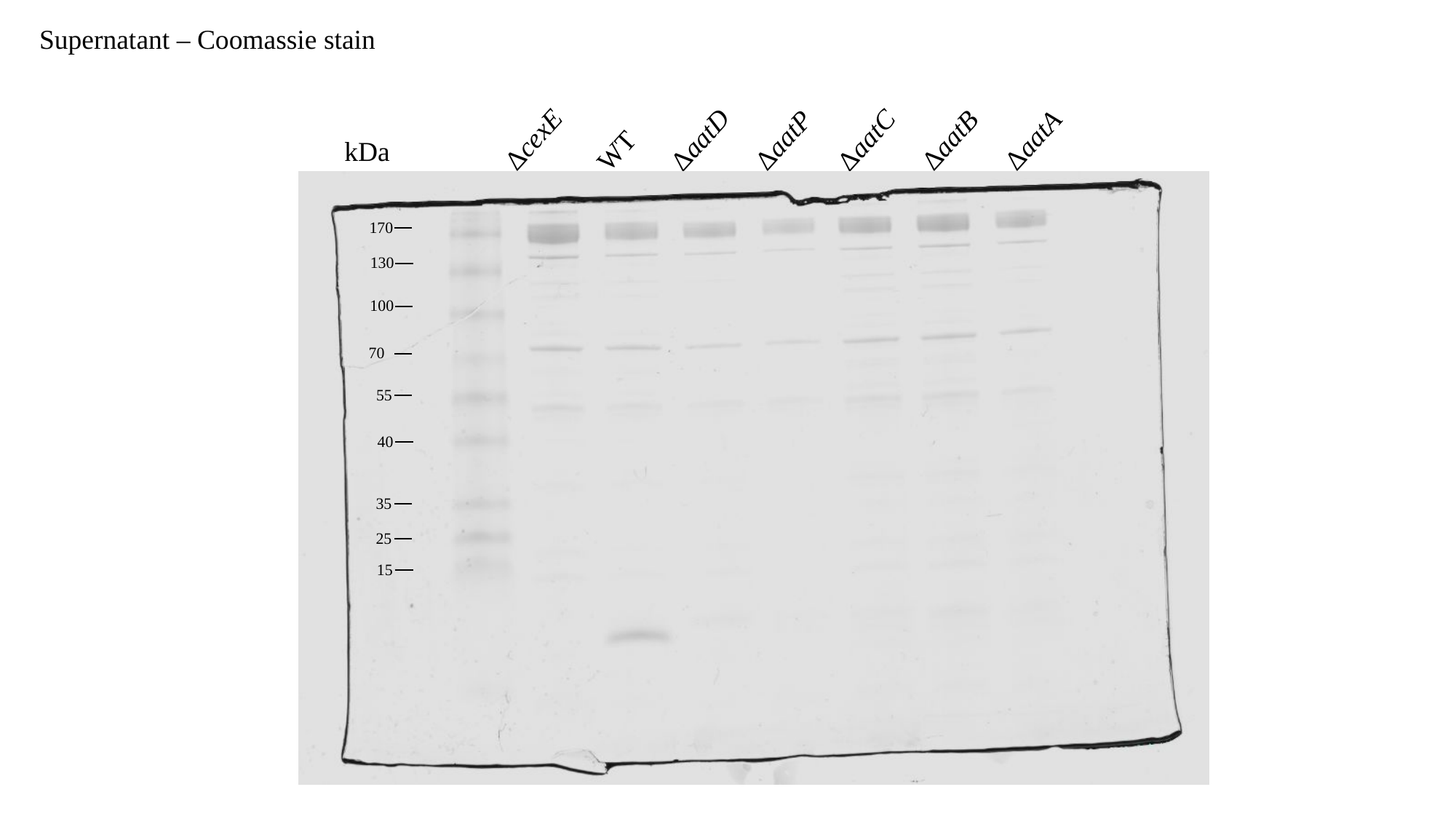

Supernatant – Coomassie stain
ΔcexE
ΔaatA
ΔaatB
ΔaatP
ΔaatC
ΔaatD
WT
kDa
170
130
100
70
55
40
35
25
15

## Slide 2
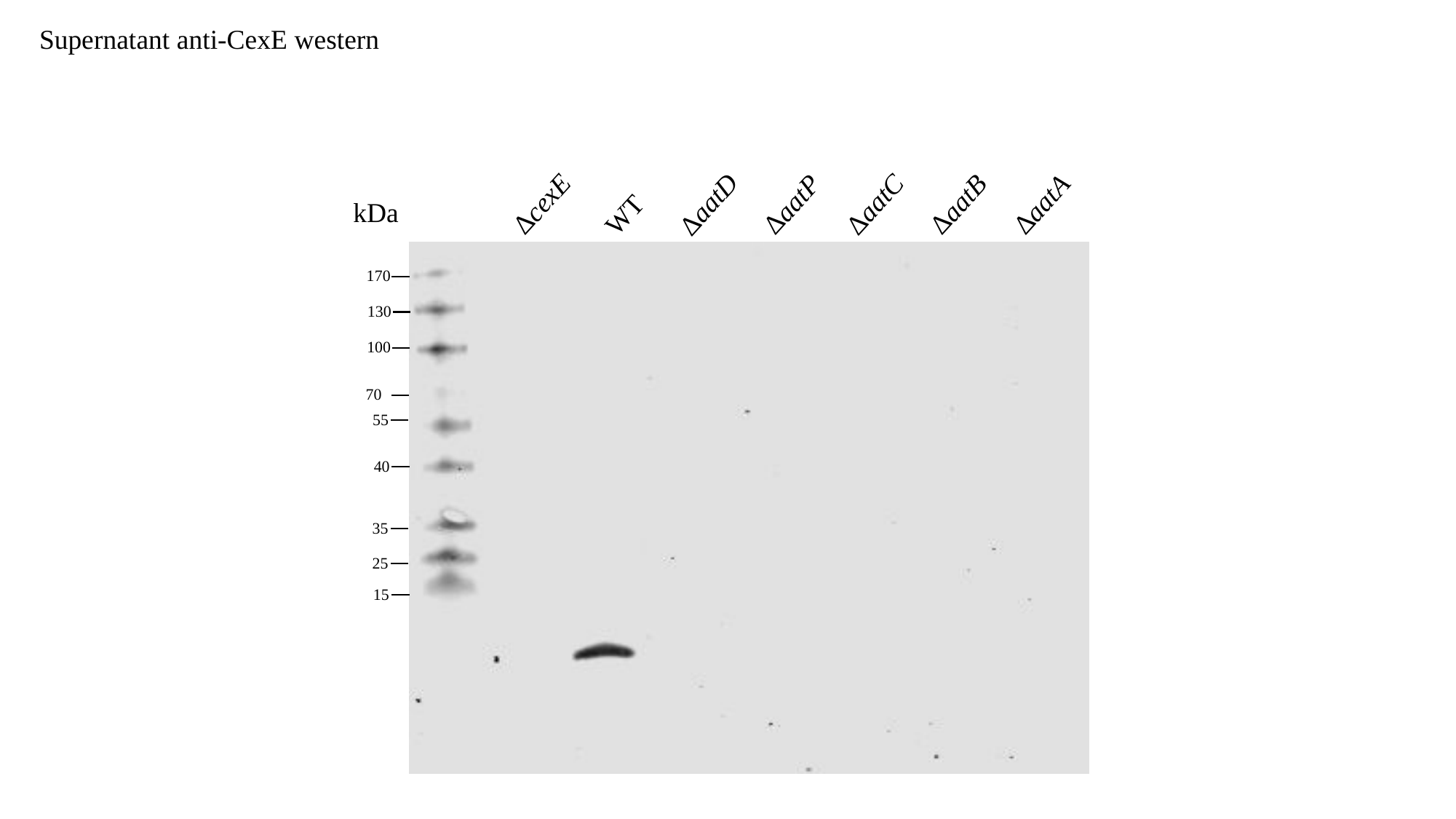

Supernatant anti-CexE western
ΔcexE
ΔaatA
ΔaatB
ΔaatP
ΔaatC
ΔaatD
kDa
WT
170
130
100
70
55
40
35
25
15
