# Supplementary figures and images for: Glycine acylation and trafficking of a new class of bacterial lipoprotein by a composite secretion system
Source: eLife. 2021 Feb 24;10:e63762. doi: 10.7554/eLife.63762 (PMC7943197; doi:10.7554/eLife.63762)

## Slide 1
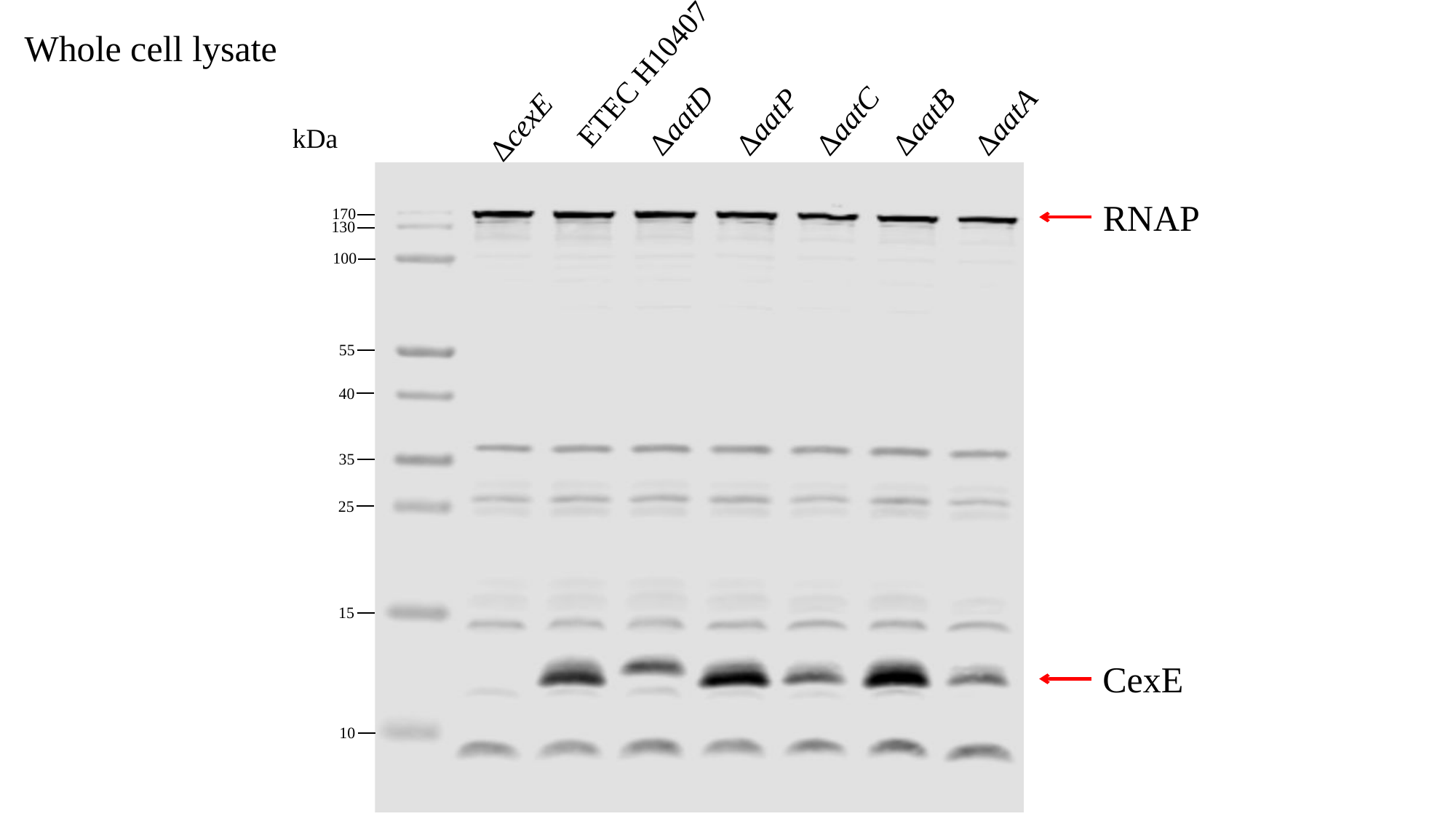

Whole cell lysate
ETEC H10407
ΔcexE
ΔaatD
ΔaatC
ΔaatP
ΔaatB
ΔaatA
kDa
RNAP
170
130
100
55
40
35
25
15
CexE
10

Supplement: Figure 2—source data 2. [file elife-63762-fig2-data2.pptx]
