# Supplementary material for: Glycine acylation and trafficking of a new class of bacterial lipoprotein by a composite secretion system
Source: eLife. 2021 Feb 24;10:e63762. doi: 10.7554/eLife.63762 (PMC7943197; doi:10.7554/eLife.63762)
Supplement: Figure 2—source data 3. [file elife-63762-fig2-data3.pptx]

## Slide 1
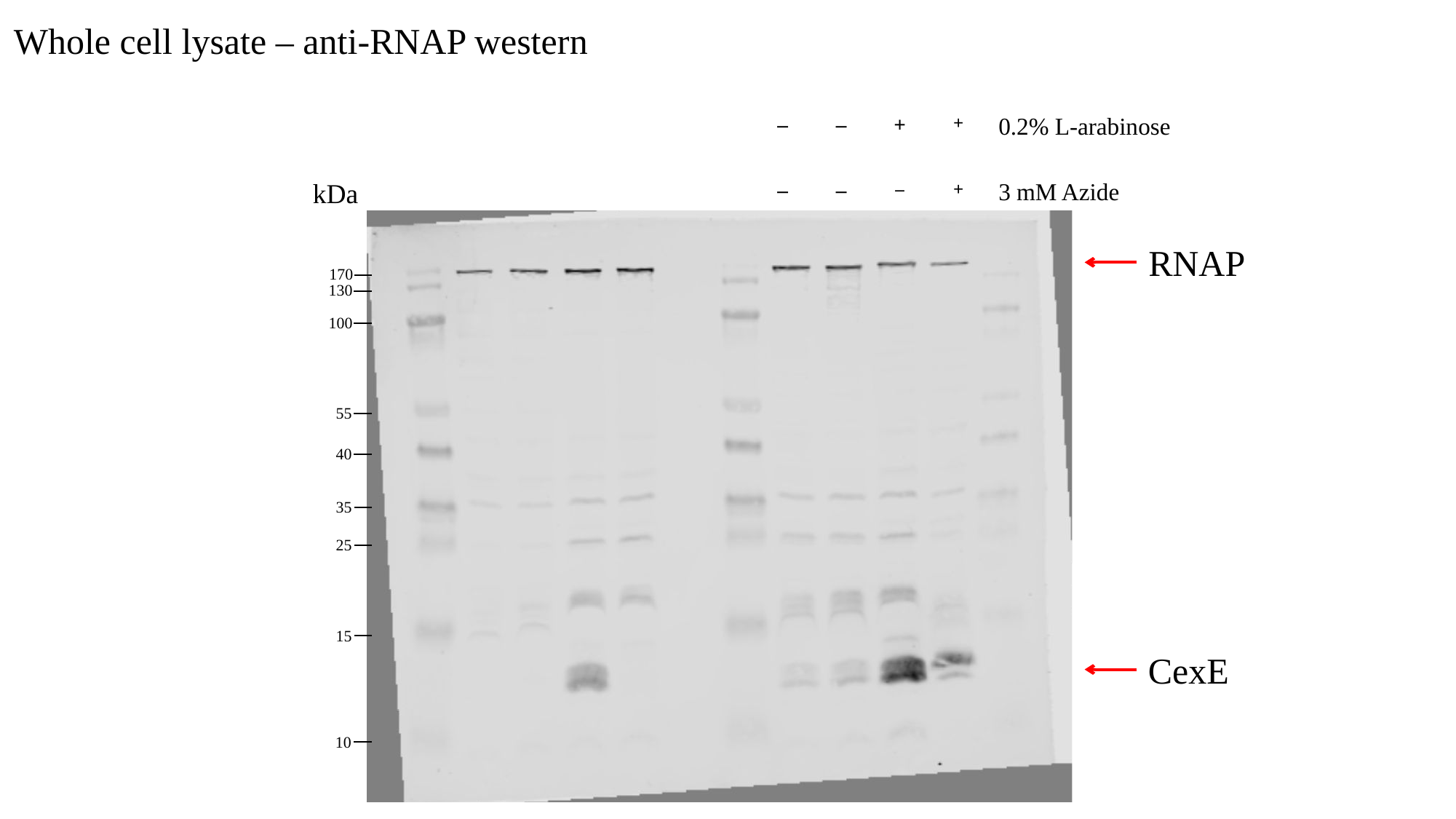

Whole cell lysate – anti-RNAP western
| ‒ | ‒ | + | + | 0.2% L-arabinose |
| --- | --- | --- | --- | --- |
| ‒ | ‒ | ‒ | + | 3 mM Azide |
kDa
RNAP
170
130
100
55
40
35
25
15
CexE
preCexE
10

## Slide 2
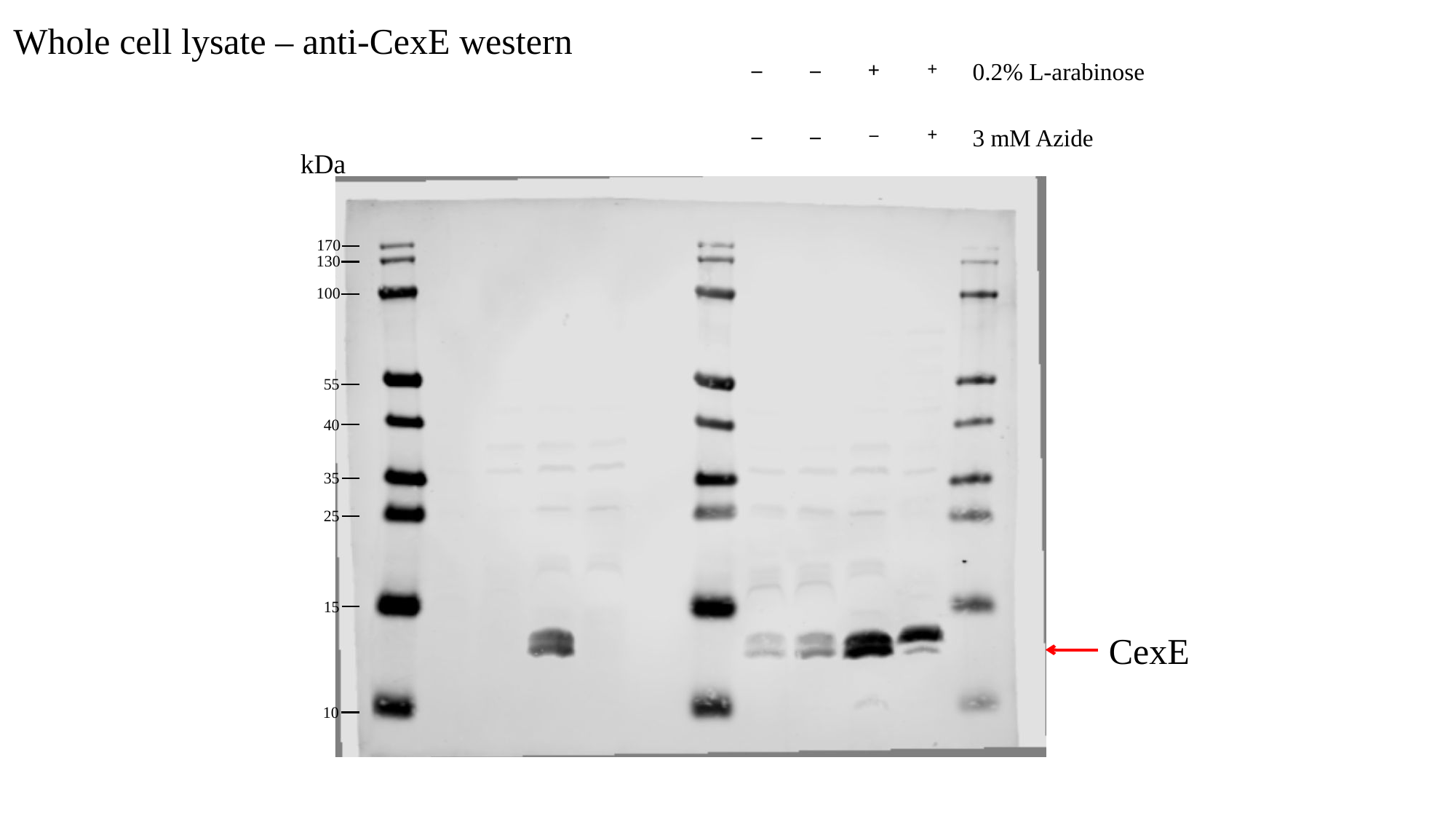

Whole cell lysate – anti-CexE western
| ‒ | ‒ | + | + | 0.2% L-arabinose |
| --- | --- | --- | --- | --- |
| ‒ | ‒ | ‒ | + | 3 mM Azide |
kDa
170
130
100
55
40
35
25
15
CexE
10
