# Supplementary material for: Glycine acylation and trafficking of a new class of bacterial lipoprotein by a composite secretion system
Source: eLife. 2021 Feb 24;10:e63762. doi: 10.7554/eLife.63762 (PMC7943197; doi:10.7554/eLife.63762)
Supplement: Figure 2—source data 4. [file elife-63762-fig2-data4.pptx]

## Slide 1
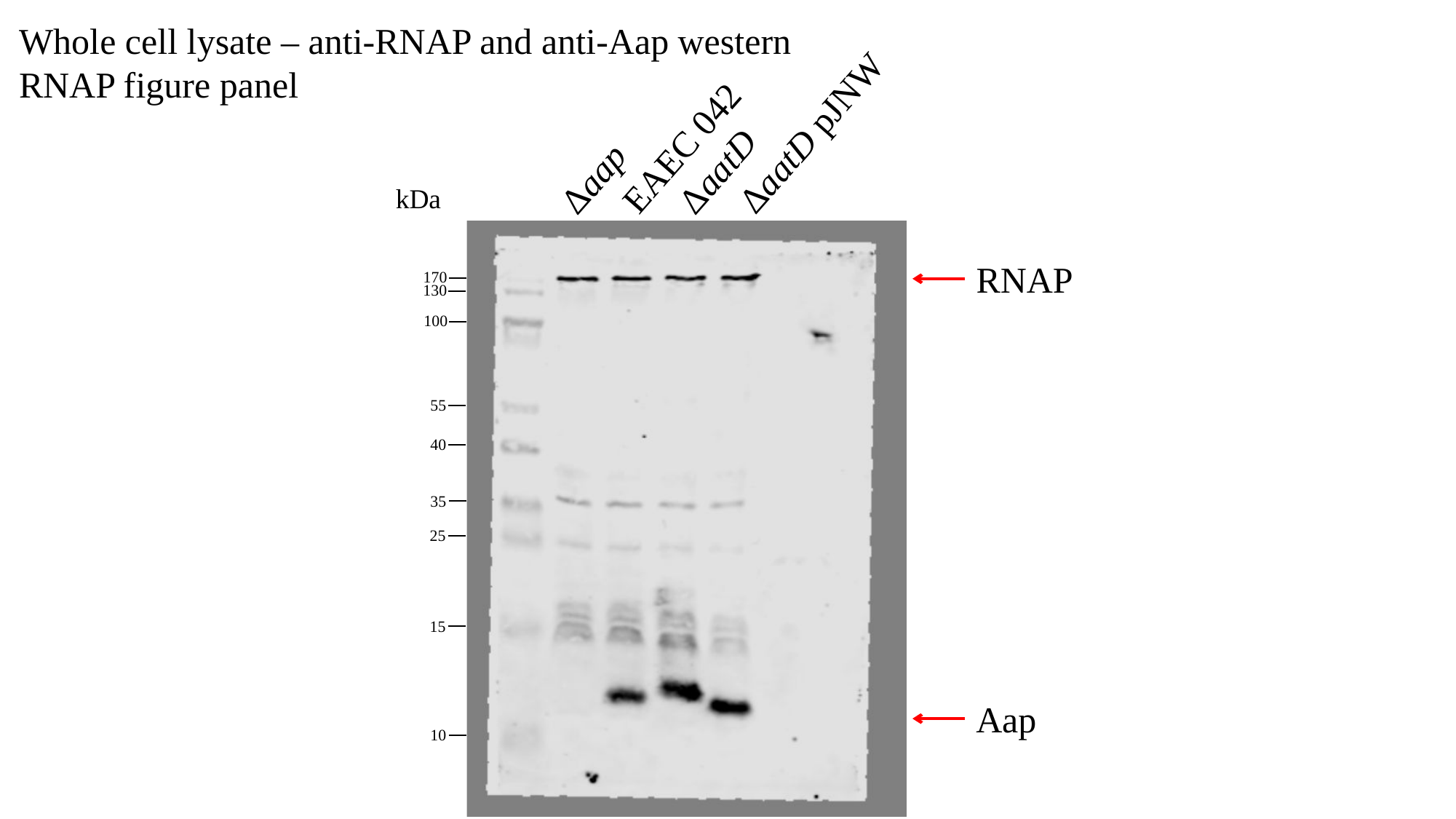

Whole cell lysate – anti-RNAP and anti-Aap western
RNAP figure panel
ΔaatD pJNW
EAEC 042
ΔaatD
Δaap
kDa
RNAP
170
130
100
55
40
35
25
15
Aap
10
