# Supplementary material for: Glycine acylation and trafficking of a new class of bacterial lipoprotein by a composite secretion system
Source: eLife. 2021 Feb 24;10:e63762. doi: 10.7554/eLife.63762 (PMC7943197; doi:10.7554/eLife.63762)
Supplement: Figure 7—source data 5. [file elife-63762-fig7-data5.pptx]

## Slide 1
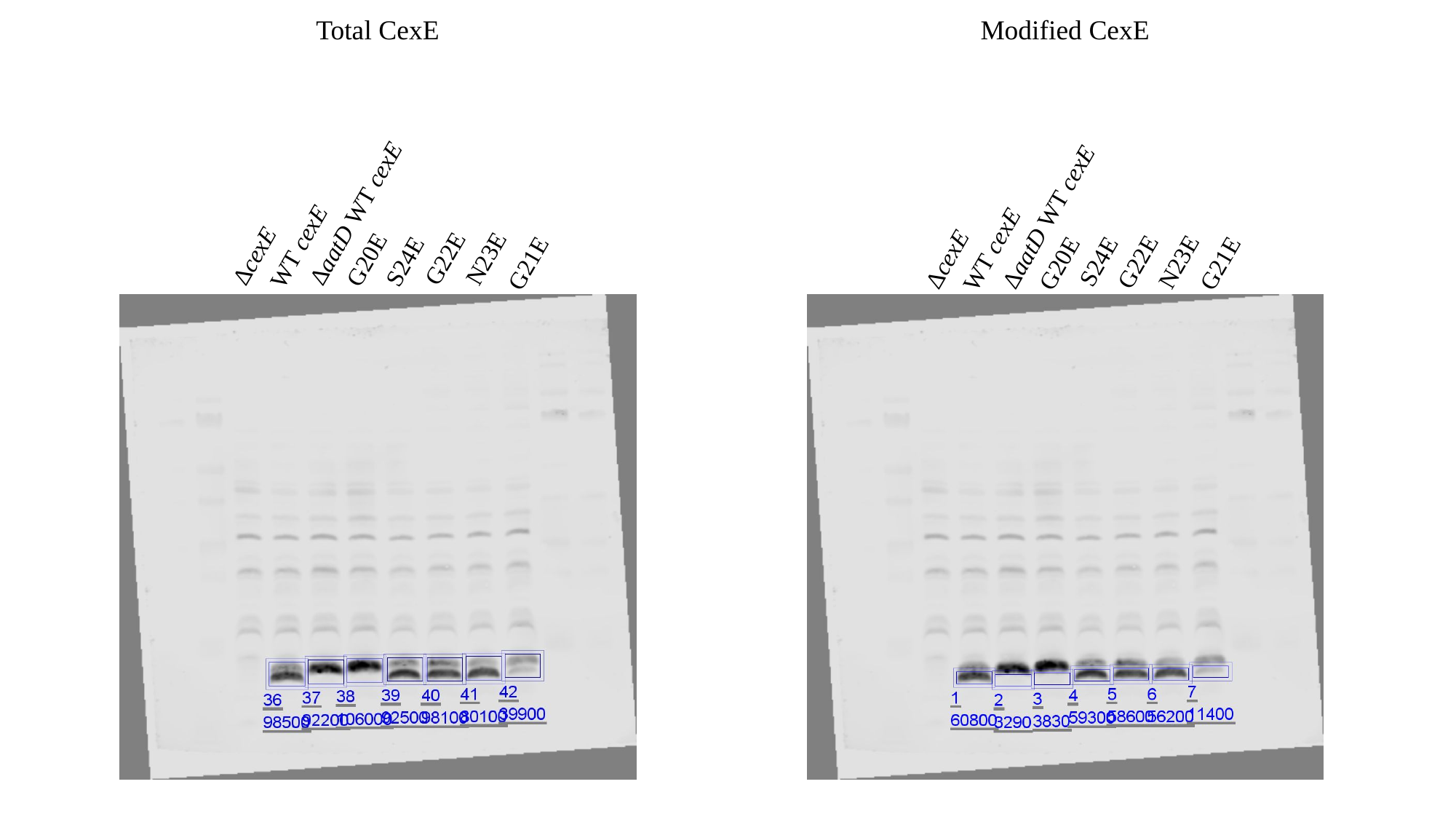

Total CexE
Modified CexE
ΔaatD WT cexE
ΔaatD WT cexE
WT cexE
WT cexE
G22E
ΔcexE
G22E
N23E
ΔcexE
N23E
G20E
S24E
S24E
G20E
G21E
G21E

## Slide 2
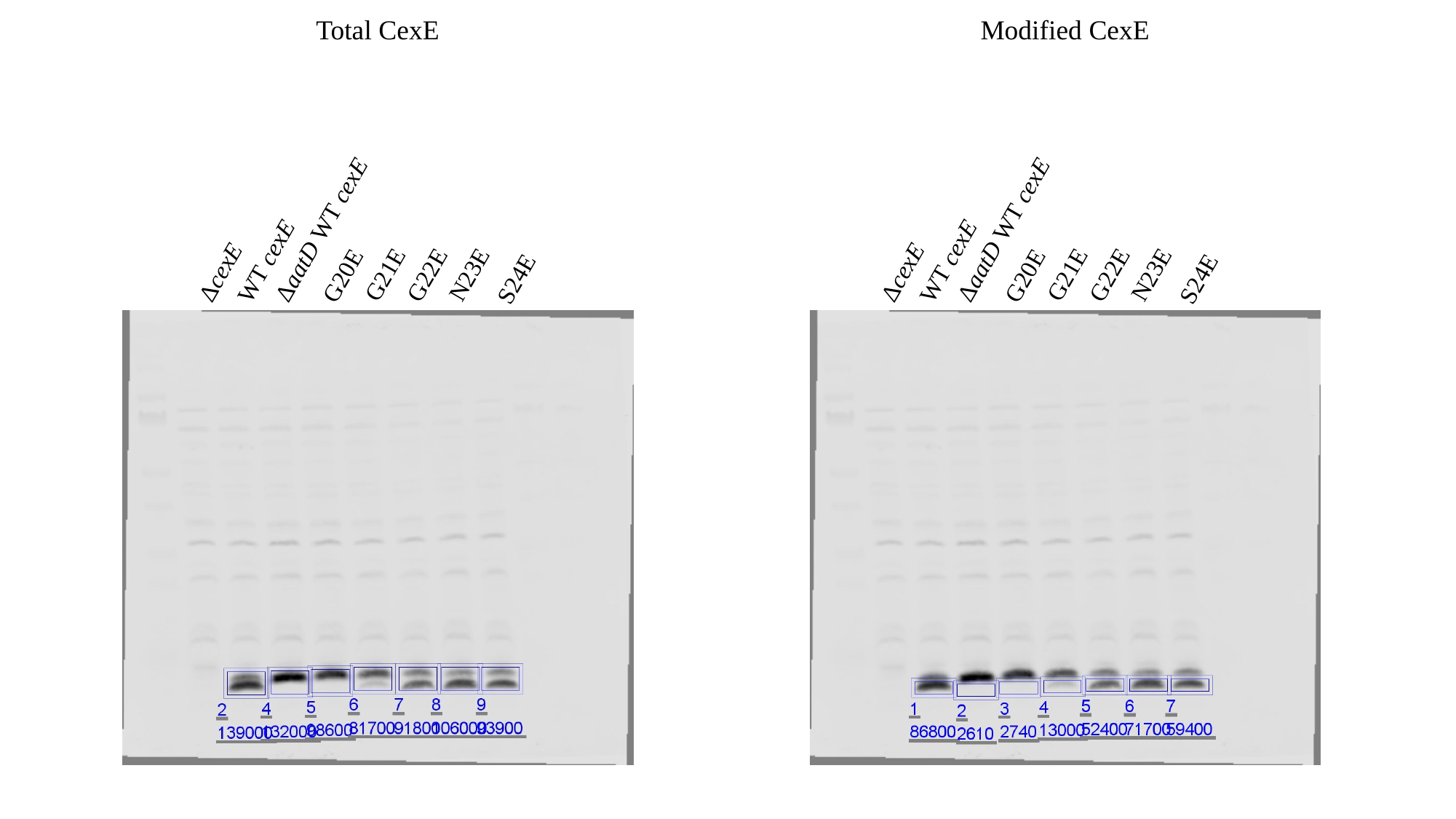

Total CexE
Modified CexE
ΔaatD WT cexE
ΔaatD WT cexE
WT cexE
WT cexE
G22E
G22E
ΔcexE
ΔcexE
N23E
N23E
G21E
G21E
G20E
G20E
S24E
S24E

## Slide 3
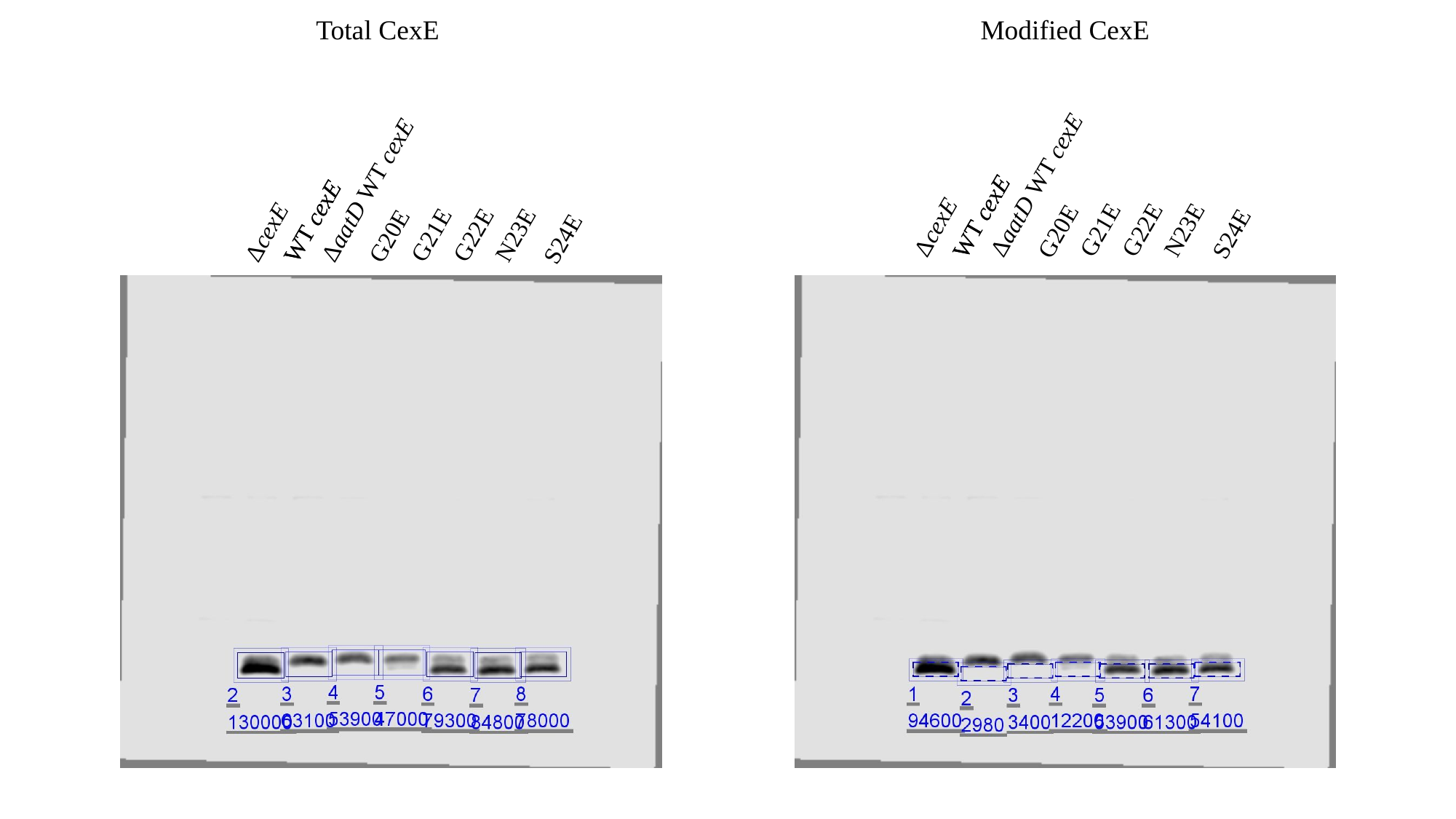

Total CexE
Modified CexE
ΔaatD WT cexE
ΔaatD WT cexE
WT cexE
WT cexE
WT cexE
WT cexE
G22E
ΔcexE
N23E
G22E
ΔcexE
G21E
N23E
G20E
S24E
G21E
G20E
S24E
