# Supplementary material for: Glycine acylation and trafficking of a new class of bacterial lipoprotein by a composite secretion system
Source: eLife. 2021 Feb 24;10:e63762. doi: 10.7554/eLife.63762 (PMC7943197; doi:10.7554/eLife.63762)
Supplement: Figure 7—figure supplement 2—source data 1. [file elife-63762-fig7-figsupp2-data1.pptx]

## Slide 1
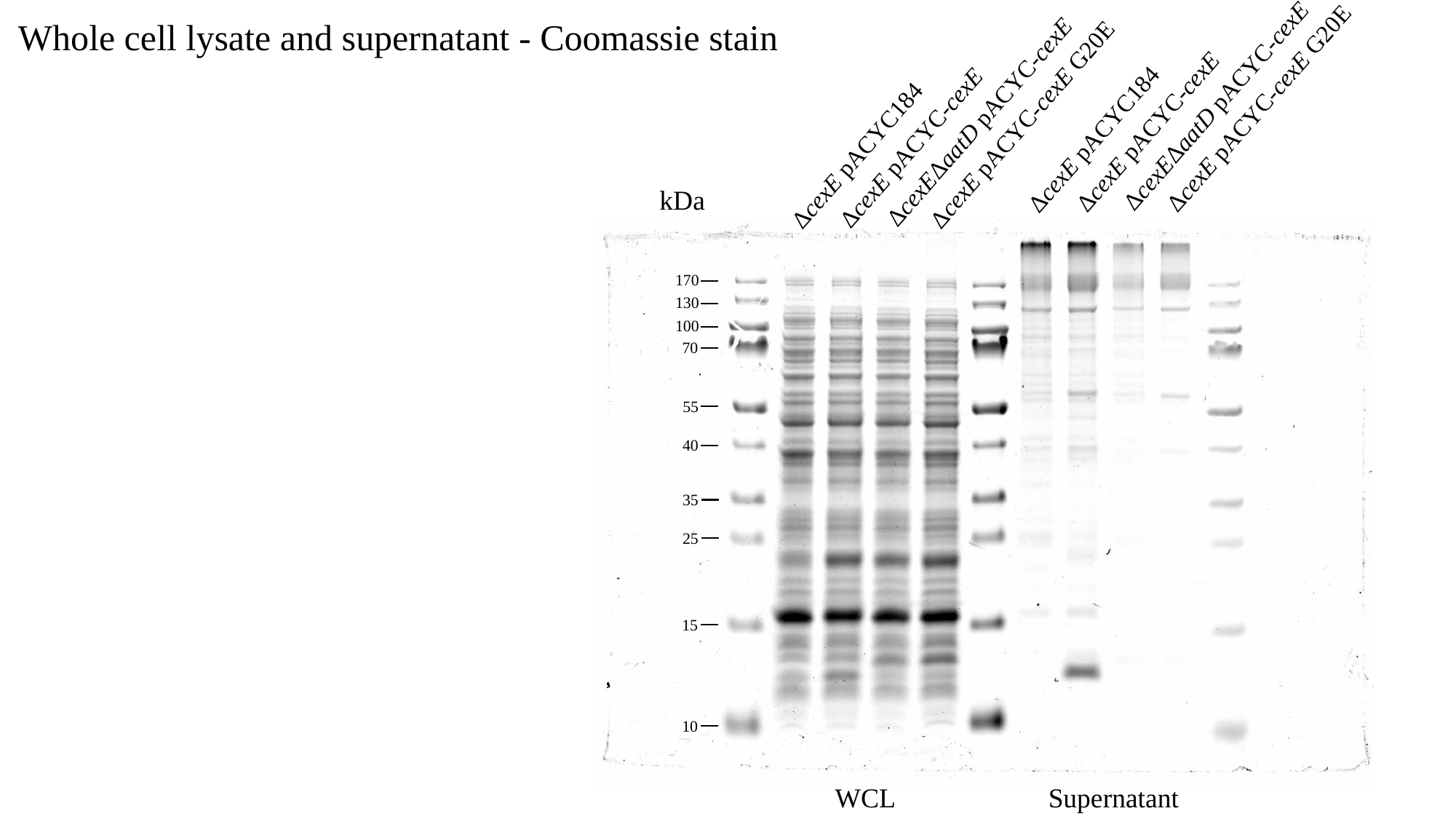

Whole cell lysate and supernatant - Coomassie stain
ΔcexEΔaatD pACYC-cexE
ΔcexE pACYC-cexE G20E
ΔcexEΔaatD pACYC-cexE
ΔcexE pACYC-cexE G20E
ΔcexE pACYC-cexE
ΔcexE pACYC184
ΔcexE pACYC-cexE
ΔcexE pACYC184
kDa
170
130
100
70
55
40
35
25
15
10
WCL
Supernatant

## Slide 2
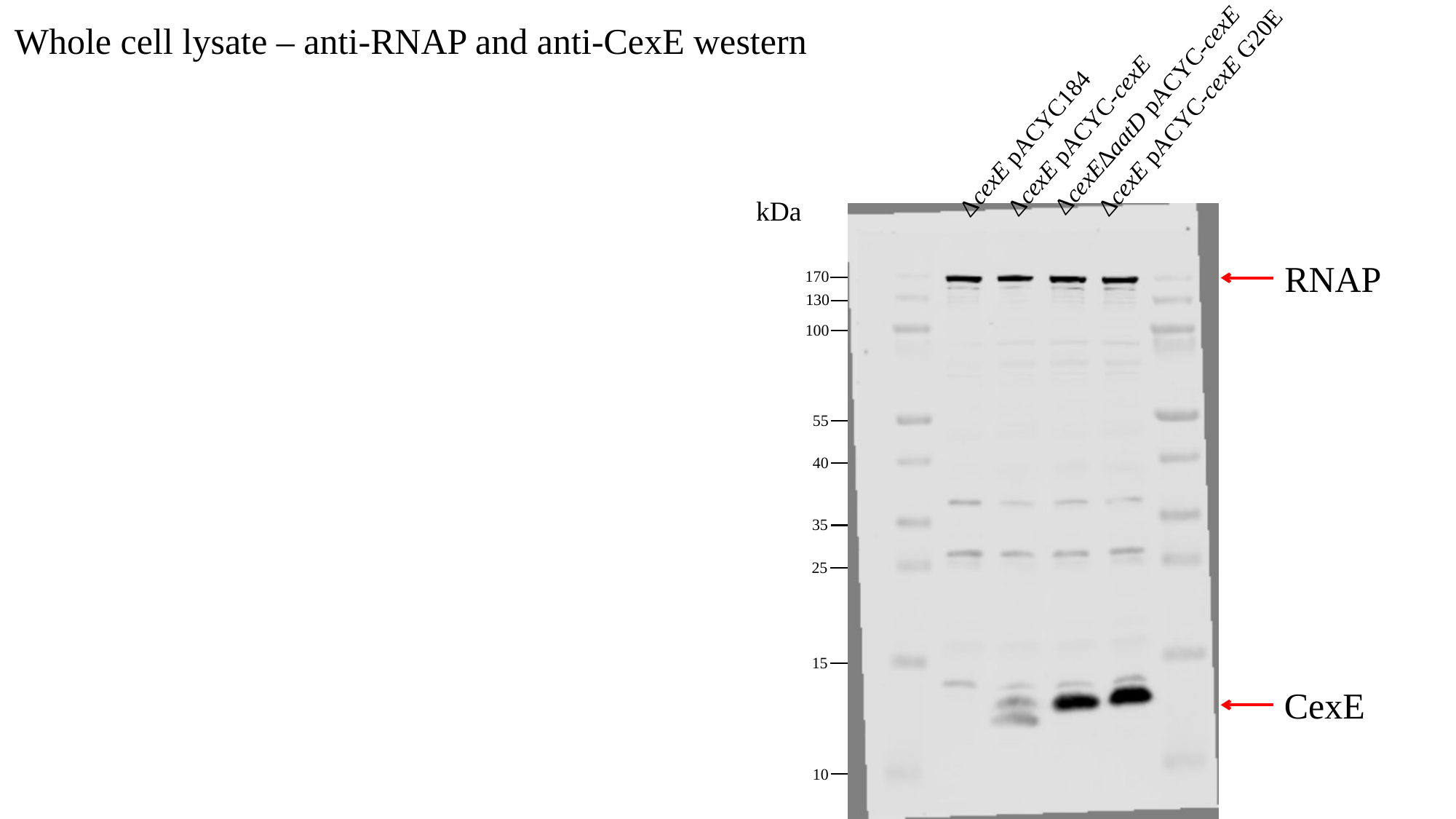

Whole cell lysate – anti-RNAP and anti-CexE western
ΔcexEΔaatD pACYC-cexE
ΔcexE pACYC-cexE G20E
ΔcexE pACYC-cexE
ΔcexE pACYC184
kDa
RNAP
170
130
100
55
40
35
25
15
CexE
10

## Slide 3
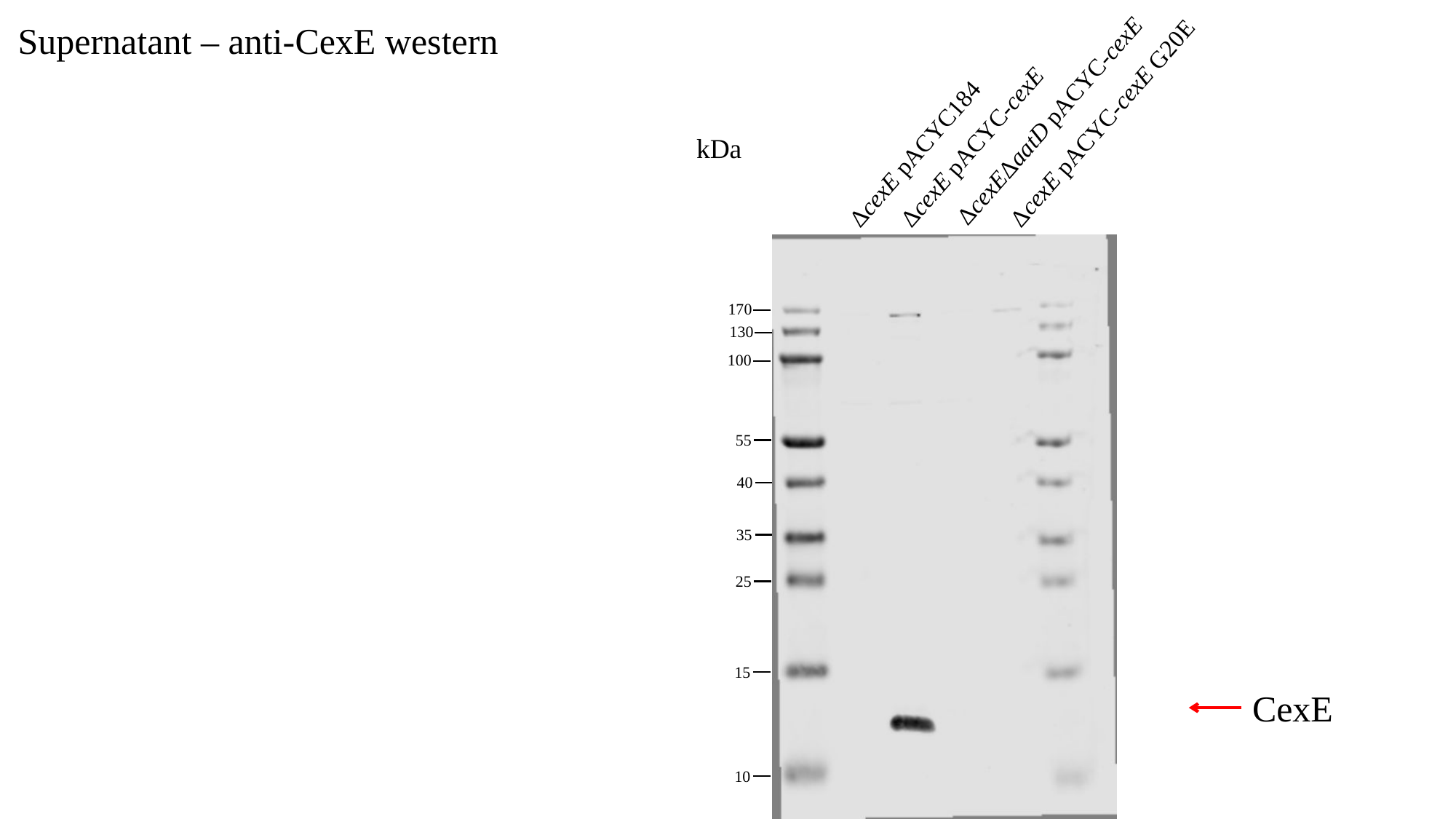

Supernatant – anti-CexE western
ΔcexEΔaatD pACYC-cexE
ΔcexE pACYC-cexE G20E
kDa
ΔcexE pACYC-cexE
ΔcexE pACYC184
170
130
100
55
40
35
25
15
CexE
10
