# Supplementary material for: Glycine acylation and trafficking of a new class of bacterial lipoprotein by a composite secretion system
Source: eLife. 2021 Feb 24;10:e63762. doi: 10.7554/eLife.63762 (PMC7943197; doi:10.7554/eLife.63762)
Supplement: Figure 9—source data 1. [file elife-63762-fig9-data1.pptx]

## Slide 1
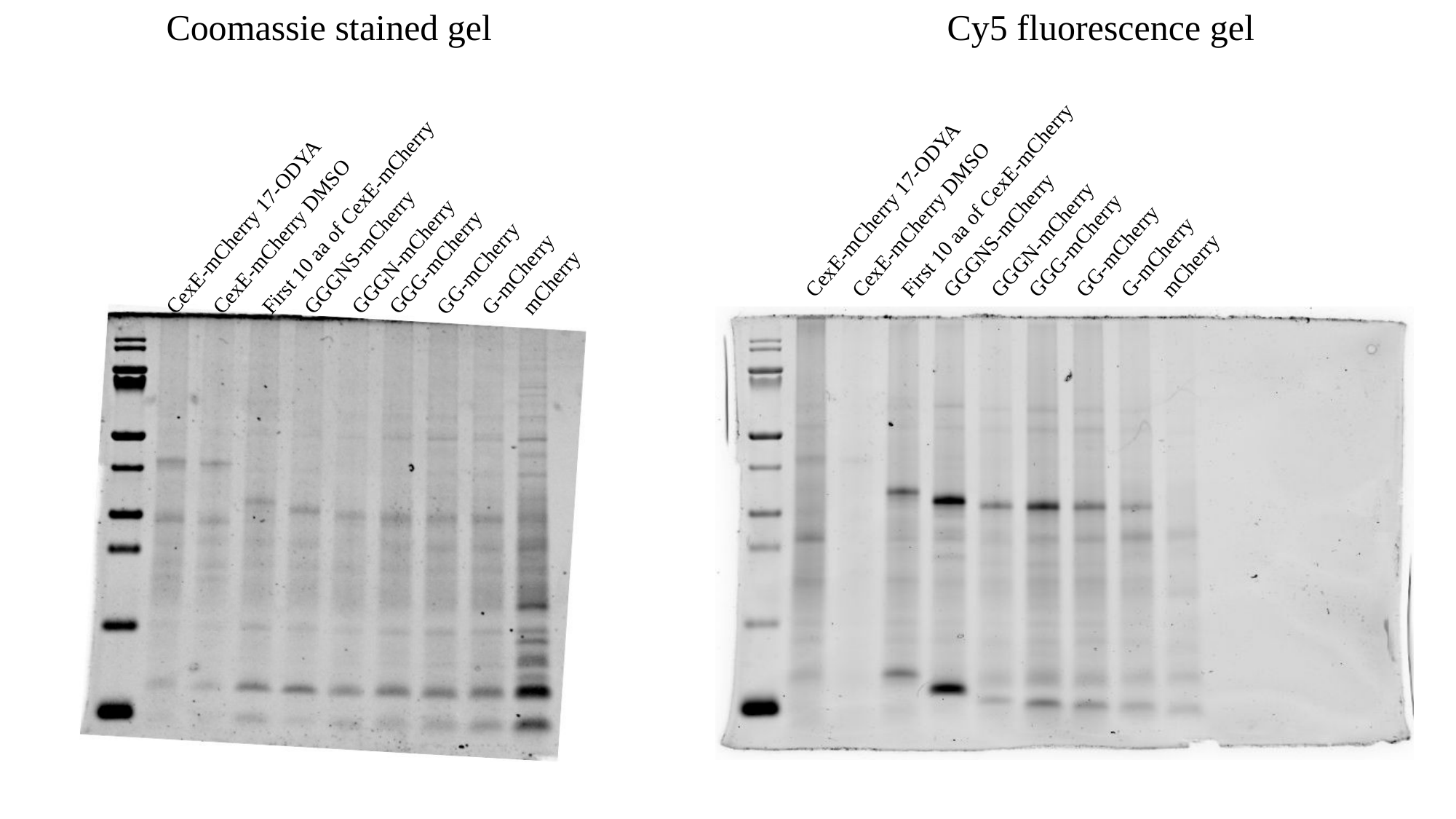

Cy5 fluorescence gel
Coomassie stained gel
First 10 aa of CexE-mCherry
CexE-mCherry 17-ODYA
First 10 aa of CexE-mCherry
CexE-mCherry DMSO
CexE-mCherry 17-ODYA
GGGNS-mCherry
CexE-mCherry DMSO
GGGN-mCherry
GGG-mCherry
GG-mCherry
GGGNS-mCherry
GGGN-mCherry
G-mCherry
GGG-mCherry
mCherry
GG-mCherry
G-mCherry
mCherry
